# Molecular xenomonitoring for *Wuchereria bancrofti* in *Culex quinquefasciatus* in two districts in Bangladesh supports transmission assessment survey findings

**DOI:** 10.1371/journal.pntd.0006574

**Published:** 2018-07-26

**Authors:** Seth R. Irish, Hasan Mohammad Al-Amin, Heather N. Paulin, A. S. M. Sultan Mahmood, Rajaul K. Khan, A. K. M. Muraduzzaman, Caitlin M. Worrell, Meerjady S. Flora, Mohammed J. Karim, Tahmina Shirin, A. K. M. Shamsuzzaman, Sanya Tahmina, Audrey Lenhart, Christine Dubray

**Affiliations:** 1 Division of Parasitic Diseases and Malaria, Center for Global Health, Centers for Disease Control and Prevention, Atlanta, Georgia, United States of America; 2 President’s Malaria Initiative, Bureau for Global Health, Office of Infectious Disease, United States Agency for International Development, Washington, DC, United States of America; 3 International Centre for Diarrhoeal Disease Research, Bangladesh, Dhaka, Bangladesh; 4 Ministry of Health and Family Welfare, Communicable Disease Control, Directorate General of Health Services, Dhaka, Bangladesh; 5 Institute of Epidemiology Disease Control & Research, Dhaka, Bangladesh; University of Buea, CAMEROON

## Abstract

**Background:**

Careful monitoring for recrudescence of *Wuchereria bancrofti* infection is necessary in communities where mass drug administration (MDA) for the elimination of lymphatic filariasis (LF) as a public health problem has been stopped. During the post-MDA period, transmission assessment surveys (TAS) are recommended by the World Health Organization to monitor the presence of the parasite in humans. Molecular xenomonitoring (MX), a method by which parasite infection in the mosquito population is monitored, has also been proposed as a sensitive method to determine whether the parasite is still present in the human population. The aim of this study was to conduct an MX evaluation in two areas of Bangladesh, one previously endemic district that had stopped MDA (Panchagarh), and part of a non-endemic district (Gaibandha) that borders the district where transmission was most recently recorded.

**Methodology/Principal findings:**

Mosquitoes were systematically collected from 180 trap sites per district and mosquito pools were tested for *W*. *bancrofti* using real-time PCR. A total of 23,436 intact mosquitoes, representing 31 species, were collected from the two districts, of which 10,344 (41%) were *Culex quinquefasciatus*, the vector of *W*. *bancrofti* in Bangladesh. All of the 594 pools of *Cx*. *quinquefasciatus* tested by real-time PCR were negative for the presence of *W*. *bancrofti* DNA.

**Conclusions/Significance:**

This study suggested the absence of *W*. *bancrofti* in these districts. MX could be a sensitive tool to confirm interruption of LF transmission in areas considered at higher risk of recrudescence, particularly in countries like Bangladesh where entomological and laboratory capacity to perform MX is available.

## Author summary

To ensure elimination of lymphatic filariasis, efficient surveillance methods are needed. While some available methods rely on the detection of *Wuchereria bancrofti* microfilaria, antigen or antibody in human blood samples, molecular xenomonitoring can identify parasite DNA in vector mosquitoes. We collected the main vector of lymphatic filariasis in Bangladesh, *Culex quinquefasciatus* mosquitoes, in two districts in Bangladesh to see if *W*. *bancrofti* could be detected. One of the districts never had evidence of widespread transmission but borders another district where transmission was most recently detected. The other district had previously had *W*. *bancrofti* transmission, but after 12 rounds of mass drug administration, had been deemed to have little to no ongoing transmission. In each district, traps were set at 180 sites to collect mosquitoes. Over ten thousand *Cx*. *quinquefasciatus* mosquitoes were collected, but none of them tested positive for presence of *W*. *bancrofti*. The practice of trapping mosquitoes was feasible for the national program to execute, and the absence of infected mosquitoes suggests that parasite rates are nearing zero.

## Introduction

Lymphatic filariasis (LF), an important cause of acute and chronic morbidity worldwide, is caused by infection with the thread-like nematodes *Wuchereria bancrofti*, *Brugia malayi* and *Brugia timori*. The Global Programme to Eliminate Lymphatic Filariasis was established in 2000 by the World Health Organization (WHO) and has two objectives: (i) the interruption of LF transmission through mass drug administration (MDA) using the combination of albendazole plus diethylcarbamazine or ivermectin, or all three drugs together in specific contexts as recommended recently by WHO [1] and (ii) the alleviation of the suffering of affected populations through morbidity management and disability prevention [2]. Interruption of transmission is thought to require at least five rounds of MDA, after which national LF elimination programs conduct a Transmission Assessment Survey (TAS) to determine whether MDA can be stopped [3].

After MDA is ceased, programs must conduct surveillance to identify and respond to the possibility of re-emergence of transmission. Current WHO recommendations for post-MDA surveillance include repeating TAS twice at 2–3 year intervals after stopping MDA, and ongoing surveillance [3]. Detection of parasites in vector mosquitoes is one of the surveillance strategies that countries can consider. Molecular xenomonitoring (MX), the use of PCR to identify parasite DNA in vector mosquitoes, has previously been used for LF surveillance after cessation of MDA [4–6] to identify residual foci of transmission. It has the advantage of being non-invasive to humans and could be useful when willingness of people to be tested is an issue, especially as households (HH) that refused MDA may also refuse testing during post-MDA surveillance. However, MX requires entomological expertise and laboratories with molecular capacity.

In Bangladesh, 70 million individuals were at risk of LF before the Ministry of Health and Family Welfare (MoHFW) started its LF elimination program in 2000 [7–8]. *Wuchereria bancrofti* is the only species of human filarial worm currently known to be present in Bangladesh and the main vector is *Culex quinquefasciatus* [9]. Based on initial mapping, 19 of 64 districts were classified as endemic (baseline microfilaria prevalence between 1% and 16%) and therefore required MDA [7], which began in 2001. By 2016, all 19 districts had passed the TAS and were eligible to cease MDA activities [7]. An ongoing surveillance project was initiated in April 2014 in Panchagarh (one of the previously treated endemic districts) and in Gaibandha (a non-endemic district that had never conducted MDA). The latter district was selected because it borders a district with recent LF transmission and was considered at high risk for re-introduction. The objective of the project was to monitor *W*. *bancrofti* transmission trends through the assessment of microfilaremia (Mf), antibodies, and antigenemia among adults in these two districts. Molecular xenomonitoring [10] was implemented as a complementary strategy for identifying areas of active transmission [11]. We sought to use MX to measure if the mosquito infection rate with *W*. *bancrofti* in the two districts was less than the cut-off point of 0.25%, a threshold that has been suggested for areas where *Culex* mosquitoes are the vector [10].

## Methods

### Study site

**Fig 1:**
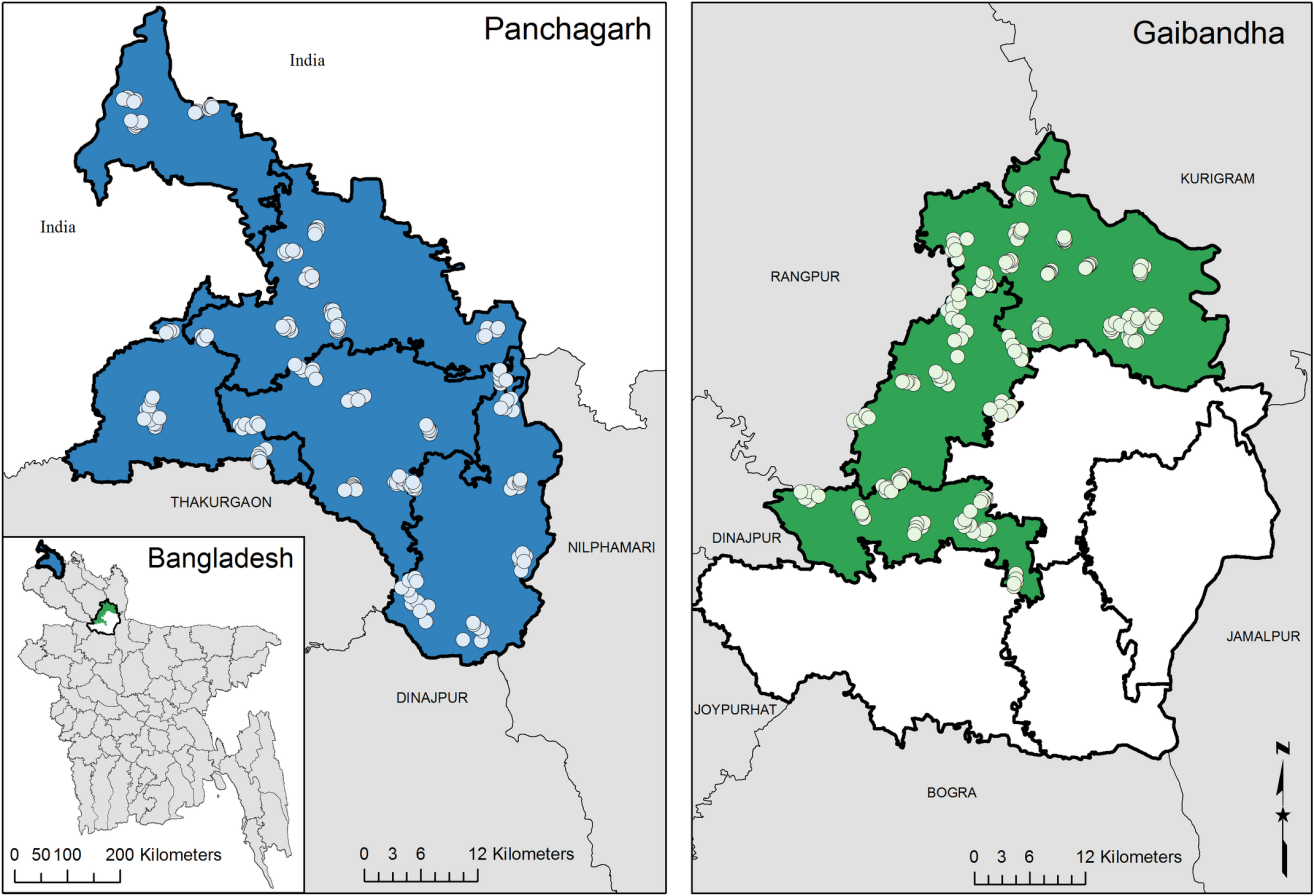
Location of the trap sites in Panchagarh and Gaibandha evaluation units.

Mosquitoes were collected in two evaluation units, one in Panchagarh district and one in Gaibandha district (Fig 1). Panchagarh district is part of the Rangpur division and is the most northeasterly district in Bangladesh, with a population of 987,644 and an area of 1404 km^2^ [12]. It is bordered on three sides by India and in the south by three other districts belonging to the Rangpur division, all of them previously endemic for LF but without any positive cases identified during the TAS1 and TAS2 (2013, 2015). The first evaluation unit in the MX study included all five sub-districts of Panchagarh district (Atwari, Tetulia, Panchagarh Sadar, Debiganj, and Boda). The LF mapping carried out in 2001 showed an LF baseline prevalence of 10.8% (Mf) in Panchagarh. MDA activities started in 2001. Following 12 rounds of MDA, the first TAS was carried out in April 2013. None of the children included in the TAS were positive for circulating filarial antigen (CFA), which made the district eligible to stop MDA. A second TAS carried out in 2015 also found no antigen-positive children [7].

Gaibandha district is also part of the Rangpur division with a population of 2.4 million and a total area of 2115 km^2^ [12]. It is bordered by six districts, three of which were previously endemic, two were classified as “low endemic”, and one as non-endemic district. One of the districts to the north-west of Gaibandha, Rangpur district, had a baseline Mf prevalence of 10.0% (2002). Rangpur did not pass the first TAS in 2013, but did pass in 2016. No MDA or TAS has been conducted in Gaibandha district, as it was not considered endemic after mapping. The evaluation unit in Gaibandha district included only three of the seven sub-districts (Palashbari, Sadullapur, and Sundarganj, population of just under 994,138, area of 785km^2^) bordering the Rangpur district. These three sub-districts were selected due to (i) the risk of LF introduction due to the proximity with the Rangpur district and; (ii) the size of Gaibandha, which was considered too large for the implementation of MX throughout the entire district.

### Mosquito sample size and sampling method

This study used a two-stage cluster sampling design. Probability proportional to estimated size (PPES) was used for the selection of villages based on the 2011 Bangladesh Census Data [12]. Thirty villages were selected for each evaluation unit. The sampling interval used to select the villages was calculated by dividing the estimated number of HHs in the evaluation unit by the number of villages (30). A number between zero and the sampling interval was randomly chosen to select the first village from the HH list, and then the sample interval was added to that number repeatedly until the 30 villages were chosen. Six HHs in each village were randomly selected (180 HHs per evaluation unit) from a numbered list of HHs provided by the village health assistants; trapping was done at these HH. CDC gravid traps were placed in each site for three consecutive nights unless more than 100 *Cx*. *quinquefasciatus* were collected prior to the third night.

The *Culex quinquefasciatus* sample size was based on a positivity threshold of <0.25%, a threshold previously suggested for areas where *Culex* mosquitoes are the vector [10]. When simple random sampling is presumed, a sample size of 6,850 *Culex* mosquitoes was required (alpha = 0.05, power = 0.75). To account for community level clustering, this sample size was multiplied by a design effect of two (13,700). This sample size was slightly decreased to 13,500 mosquitoes per evaluation unit (overall total 27,000 mosquitoes) so that it was evenly divisible by the cluster and pool size. We expected to collect at least 75 female *Cx*. *quinquefasciatus* mosquitoes over three nights at each household to be able to create three pools of 25 mosquito per trap site, which would result in a total of 540 pools of 25 mosquitoes per evaluation unit. No more than four pools were tested per site.

### Mosquito collection, identification, and preservation

Four teams of three entomologists from the MoHFW were tasked with trap deployment and mosquito identification. Each team was generally able to complete trapping in two villages per three-day collection period, resulting in collections occurring in eight villages per three-day period for the four teams.

Mosquitoes were collected using CDC gravid traps (John W. Hock Co., Gainesville, FL), which are commonly used for sampling *Cx*. *quinquefasciatus* [13]. The traps were placed within 20 meters of the selected HH. The traps, containing 2–3 day old grass infusion bait, were turned on around 18:00, and were retrieved the following morning between 08:00 and 11:00. Gravid traps and nets were labelled using stickers with barcodes. Nets containing captured mosquitoes were returned to the laboratory and placed in the freezer to kill the mosquitoes. The mosquitoes were then identified using appropriate morphological identification keys [14,15]. The sex and physiological status (unfed, fed, semigravid, gravid) was recorded for each female mosquito. Once all intact mosquitoes from a trap were identified, they were placed in a 25ml Falcon tube with silica gel, which was also labeled with a barcoded sticker. CDC gravid traps can damage some of the collected mosquitoes [13]. Only intact mosquitoes were used for the pooling for two reasons: 1) identification of *Cx*. *quinquefasciatus* in Bangladesh requires observation of features from the head, thorax, and abdomen, and 2) association of partial mosquitoes might result in mismatches which could then lead to an overestimation of infection rates if a single positive mosquito was split between two pools. After the three days of collection unfed, fed, semigravid, and gravid female *Cx*. *quinquefasciatus* from a single trap site were pooled (aiming for 25 mosquitoes per pool) and desiccated in 1.5ml Eppendorf tubes with silica gel. Unfed mosquitoes are defined as nulliparous mosquitoes, which have never fed on humans so cannot be infected, as well as parous mosquitoes, which have laid their eggs, but have not taken a next blood meal. For each trap collection site, district, sub-district, house numbers, GPS coordinates, and trap barcode numbers were entered into a data collection application developed on an Android smart phones and using the LINKS System (Task Force for Global Health, Decatur, Georgia) [16]. Data on mosquito species were recorded on paper sheets and then double-entered into Excel (Microsoft Corporation, Redmond, WA) by two people. Differences were resolved by referring to the original data sheets.

### Testing of mosquito pools

Pooled mosquitoes were tested for presence of *W*. *bancrofti* DNA at the molecular laboratory of the Institute of Epidemiology, Disease Control & Research (IEDCR, MoHFW) in Dhaka, Bangladesh. Positive and negative controls were also tested. DNA was extracted from pools using DNeasy Blood and Tissue kits (Qiagen, Hilden, Germany) following the manufacturer’s instructions. DNA quality was confirmed prior to PCR using a NanoDrop (Thermo Fisher, Waltham, MA, USA). The real-time PCR protocol described by Rao et al. [17] was used to detect *W*. *bancrofti* DNA in the pools. All reactions were carried out in an ABI 7500 Fast Dx real-time PCR system (Thermo Fisher Scientific, Waltham, MA, USA) using Taqman Universal PCR Master Mix (Thermo Fisher Scientific, Waltham, MA, USA), and all pools were run in duplicate.

### Data analysis

Data analysis was conducted using Stata 14 (College Station, TX) for descriptive statistics. The PoolScreen software (version 2.0.3) was used to determine the maximum likelihood estimate and 95% confidence intervals of *W*. *bancrofti* infection prevalence in mosquitoes [18]. The map (Fig 1) was created using ArcGIS 10.2 (ESRI, Cary, NC, USA).

### Ethics statement

This protocol was approved as a program evaluation by the U.S. Centers for Disease Control and Prevention (#2015–180). The protocol was also approved by the Bangladesh MoHFW. Written consent to place a gravid trap in the courtyard was obtained from household members.

## Results

### Mosquito collection

Mosquitoes were collected in Gaibandha district from September 28 to October 11, 2016, and in Panchagarh district from October 19 to November 1, 2016. In each site, four teams of three entomologists were needed to place the mosquito traps, collect and identify mosquitoes, and create pools. The teams took 14 days in each district to place 6 traps in each of the 30 villages and collect mosquitoes for three consecutive nights at each site. Over the course of the entire collection period 24,408 mosquitoes were collected in gravid traps; 23,436 (96%) were intact. Most mosquitoes identified to species were female (95%) and the most commonly collected species was *Cx*. *quinquefasciatus* (47%)(Table 1). A total of 10,344 female *Cx*. *quinquefasciatus* were collected during 1,079 trap nights (2 evaluation units, 180 HH per evaluation unit, 3 nights of trapping per household). For one trap location, only two nights were necessary to collect the number of mosquitoes needed. Of these, 10,021 (97%) mosquitoes were sorted into 594 pools, 267 collected in Gaibandha and 327 collected in Panchagarh (Table 2). The range of mosquitoes per pool was 1–25 (mean 16.9); and 256 pools (43.1%) were composed of 25 mosquitoes. The target of 75 mosquitoes collected per collection site was not met in 324 of 360 sites (90%); 222 of the 360 collection sites (61.7%) collected fewer than 25 *Cx*. *quinquefasciatus* over 3 nights of trapping.

**Table 1:** Total number of intact mosquitoes collected in Gaibandha (G) and Panchagarh (P) districts in September-November 2016. Mosquitoes only identified to genus are not included.

Species	Total collected	District	Female					Male
			Unfed	Fed	Semigravid	Gravid	All females	
*Culex quinquefasciatus*	10,911	G/P	1,010	142	131	9,061	10,344	567
*Armigeres kesseli*	4,068	G/P	3,658	44	35	323	4,060	8
*Armigeres subalbatus*	3,291	G/P	2,602	103	56	502	3,262	28
*Culex tritaeniorhynchus*	2,439	G/P	1,266	633	115	268	2,282	157
*Anopheles nigerrimus*	710	G/P	491	35	49	97	672	38
*Anopheles peditaeniatus*	572	G/P	528	43	0	0	571	1
*Anopheles vagus*	324	G/P	113	28	9	28	178	146
*Culex gelidus*	219	G/P	112	29	6	63	210	9
*Culex vishnui*	164	G/P	95	18	2	23	138	26
*Anopheles annularis*	141	G/P	81	18	5	17	121	20
*Culex hutchinsoni*	136	G/P	47	5	3	55	110	26
*Culex fuscocephala*	77	G/P	44	7	6	16	73	4
*Culex bitaeniorhynchus*	64	G/P	26	7	1	27	61	3
*Mansonia annulifera*	60	G/P	43	11	2	0	56	4
*Aedes albopictus*	57	G/P	48	4	0	2	54	3
*Mansonia indiana*	23	G/P	9	7	3	0	19	4
*Anopheles umbrosus*	19	G/P	14	2	2	0	18	1
*Culex pseudovishnui*	16	G/P	8	3	1	4	16	0
*Anopheles barbirostris*	15	G/P	10	2	2	0	14	1
*Mansonia annulata*	12	G/P	8	4	0	0	12	0
*Lutzia fuscana*	9	P	1	0	0	8	9	0
*Aedeomyia catastica*	7	P	3	0	0	4	7	0
*Anopheles philippinensis*	5	G	0	0	0	0	0	5
*Culex infula*	4	G	4	0	0	0	4	0
*Aedes aegypti*	3	G/P	3	0	0	0	0	0
*Ficalbia minima*	3	G	3	0	0	0	0	0
*Uranotaenia rampae*	2	P	1	0	0	1	2	0
*Coquillettidia crassipes*	1	P	0	0	0	1	0	0
*Tripteroides aranoides*	1	G	0	0	0	1	1	0
*Uranotaenia campestris*	1	P	0	0	0	1	1	0

**Table 2:** Total numbers (and interquartile ranges) of *Culex quinquefasciatus* collected in gravid traps in 1079 trap-nights and the pools created and tested for presece of *Wuchereria bancrofti* DNA.

District	Sub-district	Villages	Trap nights	Total mosquitoes	Pools tested	Complete pools (25 mosquitoes)	Positive pools
Gaibandha	Palashbari	8	144	419	51	5	0
	Sadullahapur	8	144	848	61	15	0
	Sundorganj	14	252	2832	155	78	0
Panchagarh	Atwari	4	72	474	33	10	0
	Boda	7	126	1628	84	45	0
	Debiganj	8	143	826	55	13	0
	Sadar	7	126	2563	126	84	0
	Tetulia	4	72	435	29	6	0
Total		60	1079	10,021	594	256	0

As shown in Table 1, *Cx*. *quinquefasciatus* and *Cx*. *hutchinsoni* were the only species that were predominantly collected in the gravid stage. Of the intact female *Cx*. *quinquefasciatus* collected, 88% were gravid, 10% were unfed, 1% were semigravid, and 1% were fed (Table 1). All mosquito collection data are presented in S1 Table.

### Detection of *W*. *bancrofti* in mosquito pools

None of the 594 pools tested positive for the presence of *W*. *bancrofti* DNA by PCR. Using the Clopper-Pearson method [19] in PoolScreen, the 95%CI for the infection prevalence was 0–0.00051 for Panchagarh and 0–0.00073 for Gaibandha.

## Discussion

This study describes the first MX evaluation carried out in Bangladesh during the post-MDA period to evaluate the presence of mosquitoes infected with *W*. *bancrofti*. The results showed that none of the mosquito pools tested were positive for *W*. *bancrofti* DNA. This finding correlates with results from TAS surveys carried out in the previously endemic district (Panchagarh), in 2013 and 2015 among children 6−7 years old, which did not identify any children with positive antigenemia. In the same district, the ongoing surveillance system among adults ≥ 18 years in five health facilities identified a circulating filarial antigen prevalence of less than 1% among the participants. TAS was not conducted in the non-endemic district (Gaibandha), but the routine surveillance system among adults in seven health facilities found a circulating filarial antigen prevalence of less than 1%. The sum of our MX data and recent district-level data is consistent with the absence of *W*. *bancrofti* transmission in the two districts where the MX evaluations took place.

The practical application of xenomonitoring activities is worthy of discussion. A key issue is to ensure that an adequate sample size can be attained. The main limitation encountered during the MX evaluation in Bangladesh was the difficulty in collecting sufficient mosquitoes to reach the targeted sample size of 13,500 female mosquitoes per district. Our sample size estimate was based on a on a positivity threshold of <0.25% and a design effect of two. Several sample sizes to detect culicine vector infection thresholds (all larvae stage infection) have been proposed, and range from 0.25% to 0.65%. [10, 20]. MX evaluations are labor and time intensive and a balance is necessary between programmatic feasibility and scientific rigor. In a study conducted in Sri Lanka, Rao et al. [5] evaluated a programmatically scalable xenomonitoring program in a district where they had conducted xenomonitoring previously. They estimated that 75–150 traps placed in 30 clusters to collect 300 pools (25 mosquitoes per pool) would be acceptable for programmatic implementation of xenomonitoring. Our study used approximately the same number of trap sites (180 per evaluation unit) and trap nights (3 per trap site), and the areas of Panchagarh district (1405km^2^) and the three sub-districts of Gaibandha (785km^2^) were similar in size to that of Galle district in Sri Lanka (1652km^2^). The fact that in Bangladesh we were unable to obtain the target sample size in each district is a key limitation of our study. However, if the mosquito infection rate were truly close to zero in all of the selected villages, the design effect would have been close to one. In that case, the sample size needed was half the one calculated initially (13,500), closer to the 10,021 tested in this study.

The large variation in size amongst clusters/villages also posed logistical challenges. Villages within the two study areas had sizes that ranged from two to 2875 HH according to the 2014 census. If we had calculated the sampling interval by dividing the total number of HH in the evaluation unit by the number of trap sites (180 houses per evaluation unit), some of the randomly selected villages would have received 0 traps and others 26. Instead, we calculated the sampling interval by dividing the total number of HH per evaluation unit by 30 (the total number of villages/clusters to be selected). In each of the 30 villages selected per evaluation unit, six trap-sites were systematically selected, regardless of the size of the village. By using this method, the number of traps allocated to each team and the number of villages visited daily could remain constant throughout the study.

The interpretation of zero positive pools represented a challenge in this study. LF is a focal disease, and cross-sectional cluster surveys like MX or TAS have an inherent risk of missing residual foci of transmission [21]. MX provides an indication of the potential for ongoing filariasis transmission but its most efficient use as a surveillance tool remains to be determined. For example, MX might be a complementary surveillance tool implemented in parallel with TAS for surveillance throughout an entire district (as done in this study and others [5]), with the risk of missing foci of transmission if the evaluation areas is large. A more efficient way to identify foci of transmission might be to use MX in smaller geographical areas, for example, where positive cases have been identified through a TAS [22].

In most cases, three nights were not enough to collect three pools of 25 mosquitoes per site. We wanted to undertake these collections at the time of year when the highest *Cx*. *quinquefasciatus* densities were present. However, baseline data from the study areas regarding seasonal densities of *Cx*. *quinquefasciatus* were not available, and there are differing accounts of *Cx*. *quinquefasciatus* seasonality in Bangladesh in the published literature. Begum et al. [23] found the highest densities of *Cx*. *quinquefasciatus* in December in Dhaka using human landing collections. Ameen & Moizuddin [24] found peaks in November and March, based on mosquito collections from cattle in Dhaka. Aslamkhan & Wolfe [9] found peak numbers in human landing collections in March/April in Dinajpur district, which borders Panchagarh and Gaibandha. Finally, Karim et al. [25] found the highest number of *Cx*. *quinquefasciatus* in Dhaka in the months of March and November. These data made it difficult to identify the best time of year to collect *Culex quinquefasciatus* in Gaibandha and Panchagarh. Because of budget and time constraints, we were unable to extend the collection times to ensure that three pools of 25 mosquitoes were obtained at each site. The MoHFW entomologists conducting the field work were responsible for other entomological activities in the country, ranging from sampling of malaria vectors to surveillance of *Aedes aegypti*. As such, we had to define a discrete period of collection in order for the program to adequately balance available human and budgetary resources.

The composition and maturity of the grass infusion used to bait the traps may have impacted trap yields. While grass infusion has been shown to be an effective attractant for *Cx*. *quinquefasciatus* in Tanzania [13] as well as in Dhaka during preliminary trapping, a change in the quality of the infusion was noticed over time. Drums were refilled with water and grass as soon as the previous batch was used, allowing the bacteria remaining in the drums to provide a culture for the subsequent batch. We noticed an upward trend in the number of mosquitoes collected per consecutive trap night, which might be the result of increased attractiveness of the infusion due to bacterial colonization [26]. However, as we were collecting in different villages every three nights, it is not possible to know whether this increase was due to the grass infusion or the sequence of villages where trapping was conducted. Different infusions have been used in previous studies; this likely affects catch size. For example, Rao et al. [4] used an infusion of yeast, milk powder, and dry straw. In India, a bait of hay, yeast, and water was used (S Subramanian, personal communication). It would be worthwhile to conduct an experiment to find the most attractive infusion for *Cx*. *quinquefasciatus* to standardize this aspect of xenomonitoring, while realizing that the attractiveness of these infusions may be variable from site to site. A highly attractive infusion could optimize collection efficiency and reduce the number of days of trapping needed to reach the desired sample size.

In addition to *Cx*. *quinquefasciatus*, 30 other mosquito species were collected, all of which had been previously recorded from Bangladesh [27]. Non-vectors might also be used for xenomonitoring, and testing non-vectors might help increase certainty of elimination of transmission in an area [28, 29]. Additionally, identification of all mosquito species was possible in this study because of the skill of the entomologists, but it did increase the time needed for processing mosquitoes and this capacity is not present in all locations. While our aim was to collect vectors of *W*. *bancrofti*, vectors of other diseases were collected as well. Al-Amin et al. [30] previously found malaria parasites in three of the *Anopheles* species collected in this study (*Anopheles barbirostris*, *An*. *vagus*, and *An*. *umbrosus*). *An*. *annularis* [31] and *An*. *philippinensis* [32], have also been identified as malaria vectors in Bangladesh. Of particular interest was the collection of vectors of Japanese encephalitis virus (JEV) (*Cx*. *gelidus*, *Cx*. *pseudovishnui*, *Cx*. *vishnui*, and *Cx*. *tritaeniorhynchus*). As resting collections near oviposition sites enhance the likelihood of collecting JEV vectors [33], gravid trapping might be of use for surveillance of these mosquitoes, particularly if attractants resemble their natural oviposition sites. While all species collected had been previously collected in Bangladesh, the collections of *Aedeomyia catastica*, *Armigeres kesseli*, *Uranotaenia rampae*, *Ur*. *campestris*, *Cx*. *infula*, and *Mansonia annulata* are especialy noteworthy as records of these species in Bangladesh are relatively rare [27].

Although this was the first MX evaluation carried out in Bangladesh, MX evaluations have been used in other countries to evaluate the impact of MDA on human infection prevalence [4–6, 10, 22, 34, 35]. After stopping MDA, national LF elimination programs will need to plan for post-elimination validation surveillance activities that could be routinely implemented to detect recrudescence or re-introduction of LF and to confirm interruption of transmission [36]. Though MX evaluations provide a sensitive method to detect residual foci of transmission and have been suggested by WHO as an alternative surveillance method for LF [37], not all countries have the capacity to include MX as a routine activity in their post-validation surveillance plan. MX evaluations for post-elimination validation surveillance could be recommended in high-risk transmission areas in countries with the appropriate entomological and laboratory capacities. In Bangladesh for example, where the entomological and laboratory capacity to perform MX is available, MX could be used as one of the post-validation surveillance strategies to confirm interruption of transmission in areas at higher risk of transmission identified during TAS [7]. Further operational research and information sharing about how to programmatically simplify and standardize MX evaluations will also make these evaluations more accessible to a larger number of LF endemic countries entering the post-elimination validation phase.

## Supporting information

S1 Table Mosquito collection, pooling, and village data. (XLSX)
